# Coronary circulation enhances the aerobic performance of wild Pacific salmon

**DOI:** 10.1242/jeb.247422

**Published:** 2024-07-12

**Authors:** Jacey C. Van Wert, Andreas T. Ekström, Matthew J. H. Gilbert, Brian J. Hendriks, Steven J. Cooke, David A. Patterson, Scott G. Hinch, Erika J. Eliason

**Affiliations:** ^1^ Department of Ecology, Evolution & Marine Biology, University of California, Santa Barbara, CA 93106, USA; ^2^ Department of Biological and Environmental Sciences, University of Gothenburg, 41390 Gothenburg, Sweden; ^3^ Department of Zoology, The University of British Columbia, Vancouver, BC V6T 1Z4, Canada; ^4^ Pacific Salmon Ecology and Conservation Laboratory, Department of Forest and Conservation Sciences, The University of British Columbia, Vancouver, BC V6T 1Z4, Canada; ^5^ Fish Ecology and Conservation Physiology Laboratory, Department of Biology, Carleton University, Ottawa, ON K1S 5B6, Canada; ^6^ Fisheries and Oceans Canada, Aquatic Research Cooperative Institute, School of Resource and Environmental Management, Simon Fraser University, Burnaby, BC V5A 1S6, Canada

**Keywords:** Aerobic scope, Cardiorespiratory performance, Heat tolerance, Metabolic rate, Coho salmon

## Abstract

Female Pacific salmon often experience higher mortality than males during their once-in-a-lifetime up-river spawning migration, particularly when exposed to secondary stressors (e.g. high temperatures). However, the underlying mechanisms remain unknown. One hypothesis is that female Pacific salmon hearts are more oxygen-limited than those of males and are less able to supply oxygen to the body's tissues during this demanding migration. Notably, female hearts have higher coronary blood flow, which could indicate a greater reliance on this oxygen source. Oxygen limitations can develop from naturally occurring coronary blockages (i.e. coronary arteriosclerosis) found in mature salmon hearts. If female hearts rely more heavily on coronary blood flow but experience similar arteriosclerosis levels as males, they will have disproportionately impaired aerobic performance. To test this hypothesis, we measured resting (RMR) and maximum metabolic rate (MMR), aerobic scope (AS) and acute upper thermal tolerance in coho salmon (*Oncorhynchus kisutch*) with an intact or artificially blocked coronary oxygen supply. We also assessed venous blood oxygen and chemistry (cortisol, ions and metabolite concentrations) at different time intervals during recovery from exhaustive exercise. We found that coronary blockage impaired MMR, AS and the partial pressure of oxygen in venous blood (*P*v_O_2__) during exercise recovery but did not differ between sexes. Coronary ligation lowered acute upper thermal tolerance by 1.1°C. Although we did not find evidence of enhanced female reliance on coronary supply, our findings highlight the importance of coronary blood supply for mature wild salmon, where migration success may be linked to cardiac performance, particularly during warm water conditions.

## Teaser

**Summary:** Coronary blockages impair heat tolerance and aerobic performance in wild Pacific salmon. Natural coronary blockages could thus have important implications for the conservation of migratory salmon.

## INTRODUCTION

Biological sex influences how organisms, populations and communities perform in response to environmental change (Gissi et al., 2023; Pörtner and Farrell, 2008). Performance disparities between sexes can result from differences in physiology, morphology and behavior, and are frequently more pronounced at the sexually mature life stage when species often exhibit sexual dimorphism (Hanson et al., 2008). Changes in sex-specific performance raise a potential conservation concern because maintaining functional sex ratios is crucial for sustaining a population (Kappeler et al., 2023). This is especially true for semelparous species, such as Pacific salmon (*Oncorhynchus* spp). Over the last few decades, sockeye salmon (*Oncorhynchus nerka*) have experienced female-biased mortality during their physically challenging, once-in-a-lifetime upriver spawning migration, particularly in association with additional stressors including high or low water flows, fishing interactions, handling and high temperatures (Hinch et al., 2021). There is also evidence that coho (*Oncorhynchus kisutch*) and Chinook (*Oncorhynchus tshawytscha*) salmon exhibit female biased mortality during their maturing river migration life stage (Hinch et al., 2021). Despite river migration mortality levels for female sockeye salmon being 2 to 8 times higher than those of males under stressful environmental conditions (Hinch et al., 2021), the mechanisms underpinning sex-specific mortality have not been resolved.

During their aerobically challenging spawning migration, salmon are simultaneously undergoing reproductive maturation and swimming upriver, supported exclusively by endogenous energy stores (Eliason and Farrell, 2016). Their aerobic scope (AS) represents the available aerobic capacity to support challenges like migration and is determined as the difference between resting (RMR) and maximum metabolic rates (MMR) (Farrell, 2009; Fry, 1947). During warming, blood oxygen transport increases to meet heightened metabolic demands of systemic tissues, but cardiovascular and thus metabolic performance (i.e. cardiorespiratory performance) becomes constrained at critically high temperatures in athletic fish including salmonids (Ekström et al., 2023; Eliason et al., 2013a; Farrell et al., 2009; Holt and Jorgensen, 2015). One hypothesis suggests that the constraint of cardiorespiratory performance relates to the fact that cardiac tissues become oxygen-limited at such extremes (Ekström et al., 2016; Eliason et al., 2013a; Farrell, 2009; Steinhausen et al., 2008).

The heart supplies oxygen to working tissues to support the enhanced aerobic demand during migration. The heart itself is highly dependent on aerobic metabolism to sustain cardiac function, and the oxygen supply to the heart is facilitated through two sources: the coronary artery delivers oxygen-rich arterial blood directly from the gills to the coronary vasculature of the ventricular compact myocardium, and the venous circulation delivers leftover oxygen to the ventricular spongy myocardium after the systemic tissues have first been supplied (Farrell, 2002a). The compact myocardium can range from 20 to 50% of the total ventricular mass (Brijs et al., 2017; Eliason et al., 2011), suggesting the importance of coronary circulation can vary across salmon species, populations, life-stages and sexes. The coronary artery provides distinct advantages to enhance performance (Farrell and Smith, 2017). As salmon increase their swimming speed, encounter higher temperatures or experience environmental hypoxia, there is a corresponding increase in coronary blood flow to the compact myocardium of the ventricle (Axelsson and Farrell, 1993; Ekström et al., 2017; Gamperl et al., 1995). This indicates the coronary perfusion of the heart is important during these environmental and physiological challenges. However, as adult salmon age they develop lesions from the thickening of the myointimal layer in the coronary artery (coronary arteriosclerosis) that are likely to decrease coronary blood flow (Brijs et al., 2020; Farrell, 2002b; Farrell et al., 1990). This is proposed to occur from vascular damage caused by the repeated overstretching of the coronary artery during each heartbeat (Saunders et al., 1992). While the impact of partial coronary occlusion is unknown, several studies conducting a surgical intervention to block the flow of oxygenated blood to the compact myocardium (by coronary ligation), have revealed that coronary blockage impairs cardiac conductivity during resting conditions (Brijs et al., 2020; Zena et al., 2021, 2024), and cardiac performances (e.g. stroke volume, cardiac output and ventral aortic blood pressure generation) during swimming, acute warming or environmental hypoxia in rainbow trout, *Oncorhynchus mykiss* (Ekström et al., 2017, 2019; Morgenroth et al., 2021; Steffensen and Farrell, 1998). In addition, coronary ligation reduced the critical thermal maximum (CT_max_) (Ekström et al., 2017, 2019; Morgenroth et al., 2021), functional thermal maximum (FT_max_) (Ekström et al., 2023), cardiac and aerobic scope (Ekström et al., 2018), and maximum sustained swimming speed (*U*_crit_) (Farrell and Steffensen, 1987) in salmonids.

We hypothesise that coronary ligation may have a bigger impact on the cardiorespiratory performance of females than males. Females have smaller ventricles (Clark et al., 2008; Little et al., 2020b) and a higher resting heart rate compared with males (Sandblom et al., 2009), suggesting that females may have diminished cardiorespiratory capacity compared with males. Females also accumulate more cardiac lactate than males do following handling stress (sockeye salmon; Eliason et al., 2020), but this is probably not due to an impaired *P*v_O_2__ supply to the spongy myocardium (coho salmon; Little et al., 2023). The lower activity levels of cardiac lactate dehydrogenase in females may limit their ability to cope with hypoxia and metabolize lactate (coho salmon; Little et al., 2020b). Females have higher coronary blood flow, yet at high temperatures, they are unable to increase coronary blood flow to the same extent as males (rainbow trout; Ekström et al., 2017). During a swim test where the temperature was ramped up, Ekström et al. (2023) found that female coho salmon generally had lower cardiac output, stroke volume and *Ṁ*_O_2__ compared with males. Females with coronary ligations had lower *P*v_O_2__ compared with coronary-ligated males when they quit swimming (FT_max_; Ekström et al., 2023). Thus, females may be more constrained in terms of their cardiorespiratory performance because of coronary limitations. Specifically, there may be an oxygen limitation to the compact myocardium that is potentially causing increased female mortality (Eliason et al., 2023). It remains unknown whether wild female salmon rely more heavily on coronary circulation for basic cardiorespiratory performance compared with males. The rainbow trout in Ekström et al. (2017) were of hatchery origin and may have fundamental morphological and physiological differences (Gamperl and Farrell, 2004). The wild coho in Ekström et al. (2023) underwent an experiment with the combined stress of forced swimming and warming water, and various cardiorespiratory metrics were sampled. However, the importance of coronary supply to support basic metabolism and exercise recovery across sexes remains undiscerned.

Building upon prior research using the same population as Ekström et al. (2023), we examined the role of coronary circulation on sex-specific performance, to determine whether male and female coho salmon differ in their reliance on coronary oxygen supply to support cardiorespiratory aerobic performance during exercise and recovery, or rapid environmental warming. We measured aerobic performance (RMR, MMR, AS), recovery post-exercise (excess post oxygen consumption; EPOC), the partial pressure of oxygen in venous blood (*P*v_O_2__), as well as cortisol, ions and metabolite concentrations during short-term recovery, and the acute upper thermal tolerance in salmon that were either coronary-ligated (no coronary blood flow to the heart) or sham-operated (blood flow intact). We hypothesized that ligation of the coronary artery would reduce aerobic performance, impair recovery and lower thermal tolerance across both sexes, but that this would be exacerbated for female salmon.

## MATERIALS AND METHODS

### Fish collection and holding

Adult coho salmon [*N*=41 (*n*=22 female, 19 male), fork length=55.70±0.83 cm, mean±s.e.m.; Table 1] were dip-netted at the Chilliwack River Hatchery on 30 Sep, 7 Oct and 24 Oct, 2019. These fish had recently completed their upstream migration (daily average water temperature=15.5°C) from the ocean preparing to spawn but had not fully completed sexual maturation as evidenced by their silver colouration. The fish were transported by truck 20 km within a holding tank (2700 l, stocking density ≤15 fish, >90% water air saturation) to the Fisheries and Oceans Canada Cultus Lake Salmon Laboratory in Chilliwack, British Columbia, Canada, where they were transferred to outdoor circular holding tanks (5.3 m diameter, 8000 l; stocking density ≤11 fish). These tanks were supplied with flow-through freshwater from Cultus Lake, which was filtered through sand and UV-treated, maintained at 11–12°C and >90% air saturation. A directional current was maintained in each holding tank via a submersed pump to mimic river conditions. The holding tanks had lids with transparent windows which allowed for a natural diurnal cycle. Fish were held for 1–13 days and were not fed because they naturally stop feeding during their upstream migration. All experimental protocols were approved by the University of British Columbia Animal Care Committee (#A17-0160).

**Table 1.**
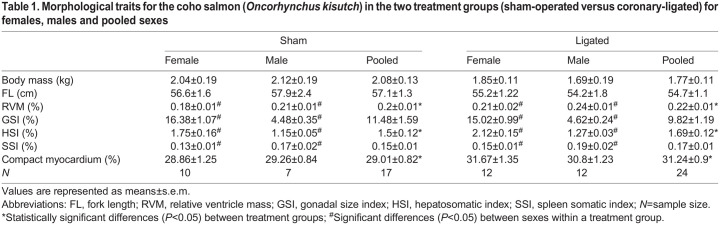
Morphological traits for the coho salmon (*Oncorhynchus kisutch*) in the two treatment groups (sham-operated versus coronary-ligated) for females, males and pooled sexes

### Surgical procedure

The surgical interventions were performed the day before the respirometry experiment. Fish were anesthetized in 12°C freshwater containing MS-222 (Tricaine methanesulfonate, 150 mg kg^−1^, buffered with NaHCO_3_, 300 mg kg^−1^) before measuring body mass. The fish was placed on its side on wet foam and a lower dose of anaesthesia (MS-222, 75 mg kg^−1^, buffered with NaHCO_3_, 150 mg kg^−1^) at 12°C continuously irrigated the gills. Fish were randomly assigned to one of two treatments: ‘coronary-ligated’ or ‘sham-operated’ (control). In both treatments, a small incision was made in the isthmus (Farrell and Steffensen, 1987). In the coronary-ligated group, the coronary artery was dissected free and ligated by tying a 6-0 silk suture around the artery, permanently restricting coronary blood flow to the ventricle. In the sham-operated group, the procedure was identical except that no suture was tied around the coronary artery. In all fish, a PE-50 cannula filled with heparinized saline (0.9% NaCl, 150 IU ml^−1^) was inserted into the sinus venosus to allow for the sampling of venous blood throughout the experiment (Ekström et al., 2023). The cannula was secured along the side and dorsal ridge posterior to the dorsal fin using 2-0 silk sutures. The fish was then placed in a recovery tank (1.95 m diameter, 1970 l, stocking density ≤2 fish) and allowed to recover overnight (12°C, >90% air saturation).

### Experimental protocol

The experimental protocol began the following day at 13:00 h, at which time individual fish (*n*=4) were transferred to a circular exercise tank (1.8 m diameter, 2000 l) receiving a high flow-through of water maintained at 12°C. Fish were manually ‘chased’ to elicit burst swimming for three min until exhaustion (Gale et al., 2014) and then immediately placed in custom-built respirometers (54.5 l) (see Van Wert et al., 2023 for respirometer details) in a flow-through experimental tank (1.8 m diameter, 2000 l). The chase protocol for this population of coho salmon has been shown to elicit a similar MMR response compared with that acquired when swimming fish to exhaustion in a swim tunnel (Little et al., 2020a).

Upon transfer to the respirometer, the cannula suture along the dorsal fin was carefully cut and the cannula was wired through a ‘snorkel’ on the top of the respirometer. The respirometer lid was sealed shut and the oxygen consumption rate (*Ṁ*_O_2__) was measured for 4–6 min to estimate MMR. Additionally, a ∼0.7 ml blood sample was taken using a heparinized syringe and the collected volume of blood was replaced with a similar volume of saline (0.9% NaCl). Following the first *Ṁ*_O_2__ measurement, a pump was automated to turn on in 10 or 15 min *Ṁ*_O_2__ cycles, comprising 6–9 min flushing periods to reoxygenate the respirometer and a 4–6 min flush-off period to measure oxygen consumption. *Ṁ*_O_2__ cycles continued overnight and were adjusted to ensure O_2_ remained above 80% air saturation. A blood sample was also taken at 15 min and 60 min following the exercise, and the following morning at 08:00 h, ∼18 h since chase (‘rest’). Owing to the increased activity levels of fish during the chase and recovery, cannulas that were dislodged from some fish and fish that were bleeding (*n*=8) were euthanized and dissected as described below. The experimental tank was sheltered with a tarp to minimize external disturbances.

After approximately 20 h of *Ṁ*_O_2__ measurements, an acute thermal ramping protocol began. Water temperature was increased at 0.1°C min^−1^ until a temperature was reached at which the fish exhibited signs of loss of equilibrium [termed ‘CT_max_’; note that the ramping rate for CT_max_ varies across studies and can influence final values (Desforges et al., 2023), but for consistency in terminology with previous related studies (Ekström et al., 2017, 2019; Morgenroth et al., 2021), we opted to use CT_max_ here]. Again, because of the increased activity levels of fish during the acute thermal ramping test, cannulas were dislodged in some fish, and fish that exhibited premature bleeding from the cannula (*n*=11) were immediately euthanized and dissected as described below. A total of 22 fish (*n*=13 female, 9 male) underwent the entire acute thermal ramping test, and at this point, blood was sampled from the cannula and fish were removed from the respirometer and euthanized by cranial blow and severing of the spinal cord. Fork length, and masses of body, ventricle, liver, spleen and gonads were measured. Ventricles were then bisected from the valves to the apex and stored in 70% ethanol for later determinations of the percentage compact myocardium using methods described by Farrell et al. (2007).

### Blood metrics

*P*v_O_2__was determined immediately after each blood sample by injecting a 300 µl aliquot into a chamber with an integrated robust fiber optic O_2_ sensor connected to a FireSting O_2_ meter (PyroScience, Germany). The chamber was sealed and placed in the experimental tank. A *P*v_O_2__ value was estimated when O_2_ plateaued at ∼3 min. The blood was then re-pooled with the original sample. All blood samples were then stored on ice for a maximum of 1 h. Haematocrit was measured in duplicate and the remaining blood was centrifuged at 1200 ***g*** for 5 min to separate the plasma, which was flash-frozen in liquid nitrogen and stored at −80°C for future analyses.

Plasma samples were assessed for cortisol, lactate, glucose, K^+^ and Na^+^, in duplicate. Cortisol was measured in a FLUOstar Omega multimode microplate reader (BMG Labtech, USA) using Cortisol ELISA kits (Neogen, USA) and read for absorbance at 650 nm, followed by the addition of 50 μl 1 M HCl and measured at 450 nm. Lactate and glucose were measured using a 2300 Stat Plus glucose and L-Lactate analyzer (YSI, USA) (Farrell et al., 2001). Potassium and sodium were measured using an XP Five-channel Flame Photometer (BWB Technologies, UK).

### Data and statistical analysis

Body morphometrics including relative ventricular mass (RVM), gonadal size index (GSI), hepatosomatic index (HSI) and splenosomatic index (SSI) were calculated by: specific organ mass/total body mass×100. Percentage compact myocardium was determined as: total dried compact myocardium/total dried ventricle tissue (spongy+compact myocardium)×100.

*Ṁ*_O_2__ data were analysed in RStudio (https://posit.co/download/rstudio-desktop/) using *AnalyzeResp* (https://github.com/kraskura/AnalyzeResp_0). The mass-specific *Ṁ*_O_2_ _(mg O_2_ kg^−1^ h^−1^) was calculated from the linear decline in O_2_ concentration over the course of each measurement cycle (ΔO_2_) in the respirometer according to *Ṁ*_O_2__=(ΔO_2_×(*v*_R_–*v*_F_))/*m*, where *v*_R_ is respirometer volume, *v*_F_ is volume of the fish (l, assuming 1 kg=1 l) and *m* is the fish mass (kg). Body mass of coho was limited to a narrow range and did not significantly influence metabolic rate. Therefore, isometric metabolic scaling was used to express mass-specific metabolism. MMR was calculated from the *Ṁ*_O_2__ measurement following the exhaustive using a sliding window analysis (180 s minimum). The ≥180 s sliding window began at the start of the measurement period and moved across the measurement in 1 s increments. The steepest ΔO_2_ with an *R*^2^>0.95 was extracted as MMR (Little et al., 2020a). RMR occurred overnight and was calculated as the lowest 10% quantile of all validated *Ṁ*_O_2__ measurements with *R*^2^>0.90. Individuals were only assessed for RMR if they had at least 60 validated *Ṁ*_O_2__ measurements. All regressions were visually assessed for negative linearity. For each fish, AAS was calculated as MMR–RMR and the factorial aerobic scope (FAS) as the ratio MMR/RMR. EPOC was calculated for each fish as the difference between the area under the *Ṁ*_O_2__ curve (AUC) from MMR until *Ṁ*_O_2__ returned to RMR and the area under RMR using the spline method in *DescTools* (https://github.com/AndriSignorell/DescTools/). EPOC duration was calculated as the time until EPOC ended.

All data were statistically analysed using R version 2022.12.0+353 with a significance level of α=0.05. All metrics were tested for normality using Shapiro–Wilk test and quantile-quantile plots and for heteroscedasticity using Levene's test. Data that did not pass normality were log_10_ transformed (RMR, RVM, GSI, HSI, SSI, percentage compact myocardium, RMR, EPOC, cortisol, glucose, and *P*v_O_2_ _at CT_max_) and reassessed for normality. To compare morphometrics across treatments and sexes, we tested for differences in body mass, FL, RVM, GSI, HSI, SSI and percentage compact myocardium using ANOVAs (type II). To test the effect of coronary ligation on performance across sexes, we tested for differences in MMR, RMR, AAS and FAS using ANOVAs (type III) with the interaction between sex and ligation treatment. The interaction was not significant for these four metrics (Table S1), so sex and treatment were tested for significance without the interaction (type II). Short-term recovery data (recovery *Ṁ*_O_2__, *P*v_O_2__ and blood metrics) were non-independent across time and were analysed using repeated measures ANOVAs. Based on BIC model selection (Table S2), sex, treatment and time point were tested as fixed effects in a linear mixed model with fish ID as a random effect (Table S3). The significance of these interactions was tested using ANOVA (type III) and Tukey's HSD *post hoc* tests (*emmeans*; https://rvlenth.github.io/emmeans/). Long-term recovery data (EPOC and EPOC duration) were assessed using one-way ANOVAs with treatment as the independent variable and sex was not included as a factor due to the low sample size. To test the effect of coronary ligation on acute thermal tolerance and *P*v_O_2__, we used an ANOVA but excluded sex as a factor due to the low sample size. All ANOVA residuals were sufficiently normal.

## RESULTS

### Morphology

Body mass and fork length did not differ between treatment groups or sexes (Table 1). RVM was greater in males than females in both treatment groups (*T*=2.74, *P*=0.009), and was greater in coronary-ligated fish compared with sham-operated fish (*T*=−2.07, *P*=0.045; Table 1). In addition, females had a greater HSI than males (*T*=−6.57, *P*<0.001) and coronary-ligated fish overall had a greater HSI than sham-operated fish (*T*=−2.32, *P*=0.026; Table 1). Females had a higher GSI than males (*T*=−19.45, *P*<0.001), whereas females had a lower SSI than males (*T*=2.68, *P*=0.011; Table 1). The proportions of compact myocardium were similar across sexes (*T*=−0.22, *P*=0.828), but the percentage compact myocardium was marginally greater in coronary-ligated fish compared with sham-treated fish (31.2±0.9% versus 29.0±0.8%, respectively; *T*=−1.77, *P*=0.085; Table 1).

### Metabolic rates

**Fig. 1.**
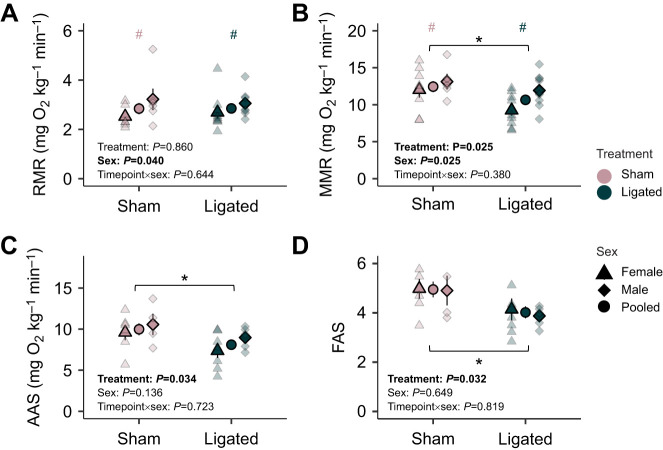
**Effects of coronary ligation on oxygen uptake rates in female and male coho salmon (*Oncorhynchus kisutch*).** (A) Resting metabolic rate (RMR), (B) maximum metabolic rate (MMR), (C) absolute aerobic scope (AAS) and (D) factorial aerobic scope (FAS). Female individuals are represented by triangles and male individuals by diamonds. The solid, larger data point denotes the mean and s.e.m. for each sex (triangles and diamonds) or pooled (circles), at each treatment (coronary-ligated versus sham-operated). *P*-values are reported for results from two-way ANOVAs with independent variables (treatment, sex) bolded if significant, with asterisks (*) for significant differences between treatments and hashtags (#) for significant differences between sexes within a treatment. See Table 2 for sample sizes.

In coronary-ligated fish, MMR was reduced by 16% (*F*_1_=5.631, *P*=0.025), AAS by 21% (*F*_1_=5.151, *P*=0.034) and FAS by 20% (*F*_1_=5.331, *P*=0.032) compared with levels in sham-operated fish (Fig. 1, Table 2). Although RMR did not differ across treatment groups (*F*_1_=0.032, *P*=0.860), RMR was statistically lower in females compared with males across treatments (*F*_1_=4.661, *P*=0.040; Fig. 1, Table 2). Additionally, MMR was significantly lower in females compared with males across treatments (*F*_1_=5.718, *P*=0.025; Fig. 1, Table 2). However, there was no significant interaction between coronary ligation treatment and sex for all metabolic metrics (Table S1).

**Table 2.**
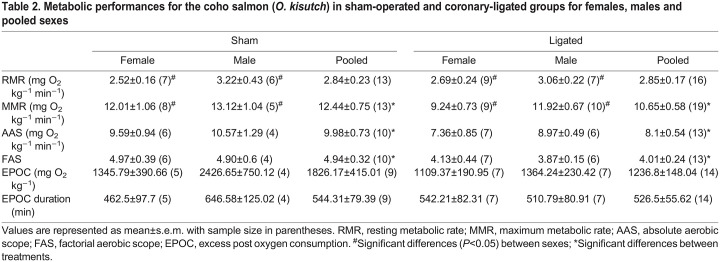
Metabolic performances for the coho salmon (*O. kisutch*) in sham-operated and coronary-ligated groups for females, males and pooled sexes

### Recovery

**Fig. 2.**
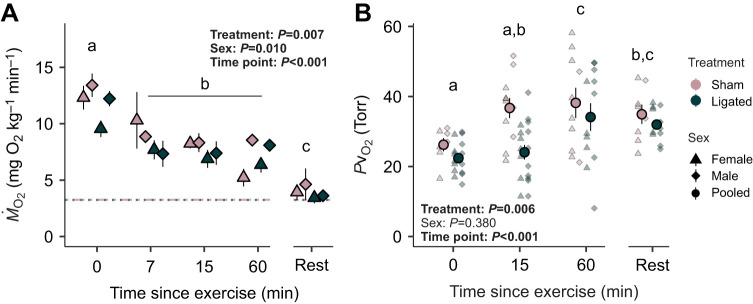
**Effects of coronary ligation on the aerobic recovery and venous oxygen supply in coho salmon (*O. kisutch*).** (A) Mean *Ṁ*_O_2__ for female (triangle) and male (diamond) sham-operated (pink) and coronary-ligated (blue) fish with the horizontal dotted lines representing the RMR for sham-operated (2.96 mg O_2_ kg^−1^ min^−1^) and coronary-ligated fish (2.92 mg O_2_ kg^−1^ min^−1^). Sample sizes at time 0: sham *N*=13 (8 female, 5 male), ligated *N*=19 (9 female, 10 male); time 7: sham *N*=6 (3 female, 3 male), ligated *N*=7 (3 female, 4 male); time 15: sham *N*=6 (3 female, 3 male), ligated *N*=8 (3 female, 5 male); time 60: sham *N*=5 (2 female, 3 male), ligated *N*=10 (4 female, 6 male); rest: sham *N*=9 (5 female, 4 male), ligated *N*=14 (7 female, 7 male). (B) The partial pressure of venous O_2_ (*P*v_O_2__) at 0, 15, 60 min, and 18 h (rest) after exhaustive exercise, with the smaller transparent points as the female (triangle) and male (diamond) individual values, and the solid, larger datapoint as the mean and s.e.m. for each treatment (sham-operated versus coronary-ligated) at every time point since exhaustive exercise. *P*-values are reported for results from linear mixed models, with individual fish as a random effect to account for repeated measures across each time point with independent variables (treatment, sex, time point) and tested for significance using repeated measures ANOVA. Values are bolded if significant. For B, values for female and male salmon are pooled within each treatment group (circle) because they are statistically the same. Different letters indicate significant differences across time points (*P*<0.05) by Tukey’s HSD.

Throughout the first hour of recovery following exhaustive exercise, *Ṁ*_O_2__ steadily declined (χ^2^=342.123, *P*<0.001) (Fig. 2A). Treatment (χ^2^=7.176, *P*=0.007) and sex (χ^2^=6.605, *P*=0.010) affected short-term *Ṁ*_O_2__, but there was no interaction between the two (χ^2^=0.248, *P*=0.618; Fig. 2). Females particularly had lower *Ṁ*_O_2__ than males (*T*=−2.419, *P*=0.020). Both time point (χ^2^=26.949, *P*<0.001) and treatment (χ^2^=7.699, *P*=0.006) had a significant effect on *P*v_O_2__ during recovery but there was no significant effect of sex (χ^2^=0.770, *P*=0.380) nor was there an interaction between these factors (χ^2^=0.525, *P*=0.469). Immediately after the exhaustive exercise, sham-operated fish had a *P*v_O_2__ of 25.2±1.9 Torr (1 Torr=133.32 Pa), whereas coronary-ligated fish had a *P*v_O_2_ _of 21.3±1.2 Torr (Fig. 2B). At 15 min into recovery, *P*v_O_2__ remained low at 23.0±2.1 Torr in coronary-ligated fish compared with sham-operated fish (35.7±2.9 Torr), which in sham-operated fish had increased to values similar to resting *P*v_O_2_ _(33.9±2.8 Torr) following full recovery (Fig. 2B). By 60 min, the *P*v_O_2__ in coronary-ligated fish had increased to nearly resting values (33.1±4.0 Torr at 60 min versus 31±1.4 Torr when fully recovered) (Fig. 2B). The long-term recovery costs (EPOC) (*F*_1_=1.197, *P*=0.286) and the full EPOC recovery durations (*F*_1_=0.036, *P*=0.852) were similar across treatments (Table 2).

**Fig. 3.**
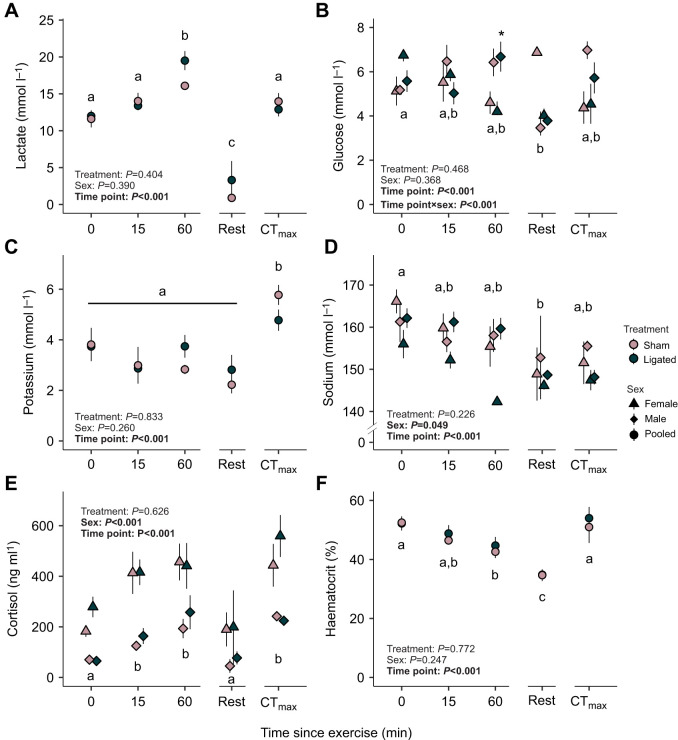
**Effects of coronary ligation on blood chemistry in coho salmon (*O. kisutch*) during recovery from an exhaustive exercise and an acute thermal ramping test.** (A) Lactate, (B) glucose, (C) potassium, (D) sodium and (E) cortisol concentrations and (F) haematocrit are represented at five experimental time points: following exercise (0, 15, and 60 min post-exercise), recovery after 18 h (rest), and after the acute thermal ramping protocol (CT_max_). Points denote the mean and s.e.m. for each treatment (sham-operated versus coronary-ligated) at every time point since the exercise with sample sizes at time 0: sham, *N*=7 (4 female, 3 male); ligated, *N*=14 (7 female, 7 male); time 15: sham, *N*=12 (8 female, 4 male); ligated, *N*=17 (7 female, 10 male); time 60: sham, *N*=13 (8 female, 5 male); ligated, *N*=16 (6 female, 10 male); rest: sham, *N*=8 (6 female, 2 male); ligated, *N*=12 (6 female, 6 male); CT_max_: sham, *N*=8 (6 female, 2 male); ligated, *N*= 13 (6 female, 7 male). Sample sizes for haematocrit values were time 0: sham, *N*=11 (6 female, 5 male); ligated, *N*=12 (5 female, 7 male); time 15: sham, *N*=12 (7 female, 5 male); ligated, *N*=13 (5 female, 8 male); time 60: sham, *N*=11 (6 female, 5 male); ligated, *N*=11 (4 female, 7 male); rest: sham, *N*=7 (5 female, 2 male); ligated, *N*=7 (4 female, 3 male); CT_max_: sham, *N*=7 (5 female, 2 male); ligated, *N*=10 (4 female, 6 male). *P*-values are reported for results from linear mixed models, with individual fish as a random effect to account for repeated measures across each time point with independent variables (treatment, sex, time point) and tested for significance using repeated measures ANOVA (Tables S2 and S3). Female and male salmon are pooled within each treatment group except for glucose, sodium and cortisol concentrations where sex or its interaction is a significant factor (females: triangle; males: diamond). Different letters indicate significant differences across time points (*P*<0.05) and asterisks (*) indicate significant differences between sexes at a time point determined by Tukey's HSD.

Plasma lactate, glucose, sodium and cortisol concentrations, as well as haematocrit significantly varied across recovery time points (0, 15, 60 min, rest) (*P*<0.001; Fig. 3; Table S3). Plasma lactate was highest at 60 min post-exercise compared with all other recovery time points (χ²=204.393, d.f.=4, *P*<0.001; Fig. 3A), with levels at 16.1 mmol l^−1^ (sham-operated) and 19.5 mmol l^−1^ (coronary-ligated). For plasma glucose, there was a significant interaction between time point and sex (χ²=41.952, d.f.=4, *P*<0.001), where males had higher plasma glucose at 60 min (*P*=0.038; Tukey's HSD). Plasma sodium concentrations varied across sexes (χ²=3.875, d.f.=1, *P*=0.049), which were generally lower in female fish compared with male fish and reached as low as 142 mmol l^−1^ in coronary-ligated female salmon at 60 min post-exercise (Fig. 3D). As expected, sex also affected plasma cortisol levels during recovery with significantly higher levels measured in females compared with males (χ²=41.528, d.f.=1, *P*<0.001; Fig. 3E). In contrast, sex did not influence lactate, potassium or haematocrit concentrations (*P*>0.001; Fig. 3; Table S3) and the ligation treatment did not impact blood metrics (*P*>0.001; Fig. 3; Table S3).

### Acute thermal limits

**Fig. 4.**
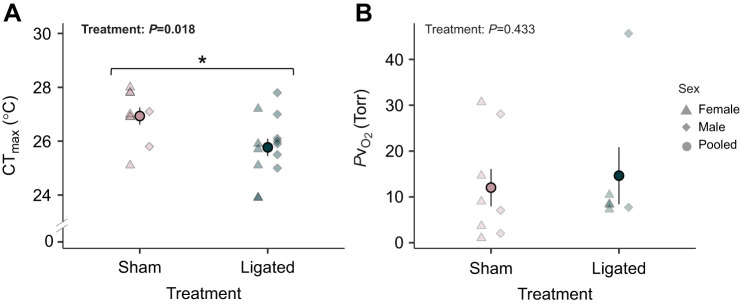
**Effect of coronary ligation on the acute thermal tolerance of coho salmon (*O. kisutch*).** Female individuals are represented by triangles, male individuals are represented by diamonds, and pooled means as circles. (A) Acute thermal limit (CT_max_) (sham: *N*=9 total, 7 female, 2 male; ligated: *N*=13 total, 6 female, 7 male) and (B) *P*v_O_2__ at CT_max_ (sham: *N*=8 total, 5 female, 3 male; ligated: *N*=6 total, 4 female, 2 male) of sham-operated and coronary-ligated salmon. Owing to low sample sizes, sex could not be included in statistical analyses and sex was pooled. The solid, circular point denotes the pooled mean and s.e.m. for each treatment (coronary-ligated versus sham-operated), and individual data points are represented for females (triangle) and males (diamond). *P*-values are reported for results from a one-way ANOVA.

Coronary ligation significantly reduced the acute thermal tolerance of coho salmon by 1.1°C from 26.9°C (sham-operated) to 25.8°C (coronary-ligated (*F*_1_=6.752, *P*=0.018; Fig. 4A). Owing to limited sample size (sham-operated, female: 7, male: 2; coronary-ligated, female: 6, male: 7), female and male thermal tolerance could not be statistically compared (Fig. 4A). The *P*v_O_2__ at CT_max_ was 12.0±4.1 Torr for sham-treated fish (*N*=8) and 14.6±6.2 Torr (*N*=6) for coronary-ligated fish and did not differ between treatments (*F*_1_=0.659, *P*=0.433; Fig. 4B). Again, because of limited sample size for females and males, the *P*v_O_2__ at CT_max_ could not be statistically compared (Fig. 4B).

After the acute thermal ramping protocol, fish had elevated lactate concentrations in both sham-operated and coronary-ligated fish compared with resting levels (14.0±1.1 mmol l^−1^ and 12.9±1.0 mmol l^−1^, compared with 0.9±0.1 mmol l^−1^ and 3.3±2.6 mmol l^−1^, respectively; Fig. 3A). Additionally, after CT_max_, potassium concentration was greater than all other time points (*P*<0.001) at ∼5 mmol l^−1^ in both treatments (Fig. 3C). Haematocrit levels after CT_max_ were also equivalent to levels reached immediately after the exhaustive exercise (*P*=0.999), nearing 55% and greater than at rest (*P*<0.001; Fig. 3F). After CT_max_, cortisol was also elevated above resting levels (*P*<0.001; Fig. 3E). Meanwhile, glucose and sodium concentrations were similar to resting values (*P*>0.05; Fig. 3B,D).

## DISCUSSION

Coronary blockage by surgical ligation impaired the aerobic performance of female salmon, but contrary to our hypothesis, female salmon do not seem to rely on the coronary supply more than their male counterparts. Rather, coronary blockage impaired aerobic performance across sexes, exhibited by reduced MMR, AAS, FAS and blood venous oxygen content during recovery from exercise. Salmon with coronary blockages demonstrated a lower acute thermal tolerance by 1.1°C compared with sham-operated fish, but owing to the limited sample size, sex-specific differences could not be discerned.

### Coronary oxygen supply is not more important in supporting female metabolism

We found a similar relative importance of coronary perfusion across sexes. This does not help to explain the high mortality of female salmon relative to males during their up-river spawning migration (Hinch et al., 2021). We still cannot discount an oxygen limitation to the female hearts as a mechanism underlying these increased mortalities, in part, because we do not have up-to-date information on the severity of coronary arteriosclerosis in wild migrating sockeye salmon. Although the severity of coronary arteriosclerosis was recorded to be similar between sexes in the 1960s (Robertson et al., 1961), the female-biased mortality emerged decades later (Hinch et al., 2021). Although females and males similarly rely on their coronary arteries, this does not discount that females could have more severe coronary arteriosclerosis and therefore less oxygen supply to the compact myocardium. Other hypothesized mechanisms also remain, such as the higher oxygen demand needed for gonad development and support in females, where males may have higher aerobic scope compared with females (Clark et al., 2011), or their differences in physiological recovery from anaerobic activity (Burnett et al., 2014; Eliason et al., 2020).

### Coronary blockage compromises metabolic performance in wild pre-spawning coho salmon

Our findings indicate that coronary ligation severely reduced MMR in both sexes, resulting in a 26% decrease for females and 10% for males. While fish were not necessarily swum to their MMR as in Ekström et al. (2023) and differences could not be discerned across sexes, sham-treated fish reached ∼12 mg O_2_ kg^−1^ min^−1^, and coronary ligated fish reached ∼7 mg O_2_ kg^−1^ min^−1^, a ∼50% difference. And at FT_max_, coronary ligation diminished *Ṁ*_O_2__ by 38%. This impairment would likely constrain their capacity to swim and endure upriver migration conditions. This finding therefore suggests that coronary arteriosclerosis may potentially result in severe consequences on the migratory capacity of salmon. Spawning Pacific salmon are recognized to develop coronary arteriosclerosis during their migration, with nearly universal incidence and up to a 48% occlusion of the coronary artery (Farrell, 2002b; Farrell et al., 1990). Theoretically, such severe blockage of the coronary artery could lead to a substantial (∼70%) decline in coronary blood flow to the heart (Brijs et al., 2020). Our findings suggest that fish with an obstructed coronary blood flow would have a lower maximal aerobic performance, which would likely compromise their swim ability during migration. This is especially important in the context of spawning migration, where some areas in the river may require maximum swimming speed, endurance or jumping capabilities (Thorstad et al., 2008) that are achieved through aerobic and anaerobic processes (Beamish, 1978). The coronary blockage in our study blocked 100% of blood flow to the coronary artery, which is more severe than the arteriosclerotic lesions observed in Farrell et al. (1990), where the artery was 48% occluded. However, those estimates came from salmon that survived the migration to the spawning grounds. It is possible that fish that did not survive the spawning migration had more severe arteriosclerosis (Farrell, 2002b).

Aerobic scope is considered to be a fundamental fitness trait and is of particular importance to migrating salmon, which presumably need 80–90% of their scope to complete their migration (Eliason et al., 2011; Farrell et al., 2008). In contrast to our prediction, aerobic scope was impacted equally across sexes. The aerobic scope in sham-treated fish matched that of a previous study on the same coho salmon population (no surgery performed), at ∼10 mg O_2_ kg^−1^ min^−1^ (Kraskura et al., 2021). However, coronary ligations reduced the aerobic scope by 19%, which corroborates previous findings in rainbow trout (*O. mykiss*) in which coronary ligation led to a 29% reduction in aerobic scope (Ekström et al., 2018). Thus, our findings suggest that cases of severe coronary arteriosclerosis will compromise coronary blood flow and impair aerobic scope, which would most likely reduce the migratory success of salmon during their once-in-a-lifetime migration (Eliason and Farrell, 2016). Furthermore, the proportion of compact myocardium (relative ventricular mass) of the coho population in this study (30%) is relatively low compared with populations with a more strenuous migration, e.g. sockeye salmon (*O. nerka*; 45% compact myocardium; see Eliason et al., 2011). Thus, populations with greater proportions of compact myocardium might rely on more coronary O_2_ supply and even with similar levels of pathology, might be even more compromised.

Direct impairment of the compact myocardium by coronary occlusion would reduce cardiac output and tissues would have to extract a greater portion of available blood oxygen to meet aerobic demands, resulting in lower *P*v_O_2__ supplied to the heart. The low *P*v_O_2__ supply to the spongy myocardium would further compromise cardiac function, and thus aerobic capacity. Indeed, during and immediately after exhaustive exercise, the *P*v_O_2__ and thus O_2_ supply to the inner spongy myocardium declined to 20–30 Torr across both treatments, which is consistent with values previously measured in fatigued salmon (Ekström et al., 2023; Eliason et al., 2013b; Farrell and Clutterham, 2003; Little et al., 2023; Steinhausen et al., 2008). In some coronary-ligated individuals, the *P*v_O_2__ reached as low as 7–10 Torr, which surpasses the threshold (19 Torr) for bradycardia and arrhythmia following exercise (Ekström et al., 2023; Wallbom et al., 2023) and the threshold (10 Torr) that induces cardiac collapse in salmonids (Clark et al., 2008; Davie and Farrell, 1991; Hanson and Farrell, 2007). In combination with the reduced *P*v_O_2__ and the need for increased cardiac output during exercise (Ekström et al., 2023), the *P*_O_2__ gradient between the luminal blood and cardiac mitochondria was likely insufficient to adequately support cardiac pumping (Davie and Farrell, 1991). According to Fick's principle, *Ṁ*_O_2__ equals the cardiac output multiplied by the difference in arterial and venous oxygen content. *Ṁ*_O_2__ levels in coronary-ligated fish did not increase above sham-treated fish with the corresponding low *P*v_O_2__ at 0 and 15 min, but this could be the result of changes in several cardiorespiratory variables. Tissues may have extracted more oxygen from the blood, leading to a greater difference between arterial and venous oxygen content, or less oxygen could have been delivered to tissues due to reduced cardiac output, leading to increased oxygen extraction and lower *P*v_O_2__. In fact, during recovery from swimming, cardiac output would be expected to decline (Eliason et al., 2013b). Prior work showed evidence that coronary ligation causes cardiac arrhythmia and bradycardia that severely constrains cardiac function and cardiovascular O_2_ transport to systemic tissues following and during exhaustive exercise, which ultimately constrains metabolic capacity (Ekström et al., 2023; Wallbom et al., 2023; Zena et al., 2024). Our current findings reinforce that during exercise, salmon are highly dependent on the delivery of oxygen to the heart via coronary circulation.

Migrating salmon use anaerobic (glycolytic) burst swimming to navigate high flows and rapids (Rand and Hinch, 1998). Given their limited energy stores and narrow time window to spawn, salmon must be able to recover in a timely and effective manner (Birnie-Gauvin et al., 2023). Fish had increased plasma lactate concentration during the 1 h post-exercise recovery period. This elevated lactate coincided with a lower *P*v_O_2_ _at 1 h in the coronary-ligated fish, suggesting that ligated fish had a greater reliance on glycolysis to meet tissue energy demands, or that the normal recycling of lactate as a fuel for cardiac metabolism was compromised, reducing lactate clearance rates. The accumulation of lactate was concurrent with declining sodium levels and elevated potassium levels, which could also be associated with extracellular metabolic acidosis, leading to impaired cardiac contractility in fish (Hanson and Farrell, 2007). Similarly, plasma lactate accumulated in swimming coho at FT_max_ and coincided with elevated potassium levels (Ekström et al., 2023). Interestingly, plasma lactate accumulated to a lesser extent in the FT_max_ coho salmon study, reaching 5 mmol l^−1^ compared with nearly 20 mmol l^−1^ here (Ekström et al., 2023). This may be due to differences in the exercise protocols used (exhaustive chase here versus swim tunnel in Ekström et al., 2023) (Milligan and Wood, 1986). Nevertheless, the coronary blockage might have impeded the capacity to sustain cardiac performance through different mechanisms that are still not entirely elucidated but are evidenced here and in previous work.

The coronary-ligated fish also had larger RVM and greater percentage of compact myocardium, which conflicts with findings that compact myocardium decreases in coronary-ligated rainbow trout after 3 days (Zena et al., 2021). However, these coho salmon may have had a varied response to coronary ligation, and the heart may have remained in the inflammatory response stage three days since coronary ligation, which is characterized by an infiltration of inflammatory cells to the damaged heart area for repair (Grivas et al., 2014; Zena et al., 2021). Fish also appeared to have responses to coronary ligation beyond the cardiorespiratory system. The liver is involved in metabolism and energy storage (Harper and Wolf, 2009), and the greater HSI in coronary-ligated fish compared with sham-treated fish indicates that the liver underwent hyperplasia or hypertrophy, a common stress response.

### Coronary circulation improves acute thermal tolerance

The performance metrics described above could be especially critical during environmental challenges, including warming water temperatures, increased or decreased flow rates, or hypoxic conditions. In our study, building upon prior research conducted with coho salmon of the same population (Ekström et al., 2023), we observed that coronary ligation lowered acute temperature tolerance by 1.1°C. Similar effects were found in rainbow trout with coronary-ligated coronary arteries (Ekström et al., 2017, 2019; Morgenroth et al., 2021). Ekström et al. (2023) swam coho salmon, from the same population as this study, in a swim tunnel to measure thermal limits (CT_swim_) and found that coronary ligation substantially lowered thermal limits by 4.4°C. This underscores the significance of coronary circulation in coping with the combination of energetic demands of intense swimming and elevated temperatures. One underlying mechanism contributing to the lower thermal tolerance in coronary-ligated rainbow trout was a decline in stroke volume and cardiac output, culminating in the failure of cardiac function at lower temperatures (Ekström et al., 2019; Morgenroth et al., 2021). At the acute thermal limits, the blood *P*v_O_2__ of both sham-operated and coronary-ligated fish neared 12–14 Torr and reached below 10 Torr for nine fish. These values nearing the 10 Torr threshold for cardiac collapse in salmonids suggest thermal tolerance was in part, limited by the heart (Clark et al., 2008; Davie and Farrell, 1991; Hanson and Farrell, 2007). We did not necessarily expect differences in *P*v_O_2__ between treatments because the blood was sampled at CT_max_ for each fish.

The CT_max_ values measured here (25–27°C) are far higher than the thermal tolerances of salmonids measured using more ecologically relevant techniques (Mayer et al., 2023). CT_max_ tests ramp temperatures at an acute rate until loss of equilibrium, thus indicating an upper thermal limit indicative of death, not functional thermal tolerance (Blasco et al., 2020; Desforges et al., 2023). Indeed, a more functional test swam fish during acute warming (CT_swim_) in the same coho population with the same sham and coronary ligation treatments and found lower functional limits, ranging from 15 to 25°C across treatments (Ekström et al., 2023). Sex-specific differences in survival and performance are revealed at temperatures below 20–21°C (Hinch et al., 2021). Current Fraser River watershed temperatures rarely exceed 22°C (Fraser River EWatch, 2023) and elevated en route mortality is observed when temperatures are >18°C for many salmon populations (Martins et al., 2012). Although the CT_max_ values do not provide ecologically relevant thermal thresholds, they can be used to compare across treatments and studies to reveal relative thermal tolerance. The mechanisms responsible for the differences in CT_max_ between the treatments may similarly impact physiological performance at lower temperatures and have long-term impacts. Therefore, care should be taken when interpreting these findings, but the implications remain.

### Concluding remarks

The productivity (e.g. recruits per spawner) of Pacific salmon has been in decline in their southern range for the past 30 years (Peterman and Dorner, 2012) with adult returns to spawning grounds at record low abundances in many populations. Those individuals that do succeed to spawn must demonstrate exceptional cardiac performance. If salmon have impaired cardiorespiratory fitness, such as reduced MMR, aerobic scope and recovery rates when coronary blood flow is restricted, they may be less likely to complete their spawning migration. This raises the question of how these effects extend to the wild and across salmonid populations and species (Farrell, 2023). Although coronary circulation is a secondary oxygen supply to the heart in certain fish, unlike its primary role in supporting hearts in birds and mammals (Farrell et al., 2012), we highlight its potential significance in wild migrating salmon. We propose that the ramifications of coronary arteriosclerosis for salmon could become more severe as they confront growing and novel challenges during their upstream migrations in the face of climate change. This underscores the value of studying heart function at the foundational level and within the framework of climate change.

## Supplementary Material

10.1242/jexbio.247422_sup1 Supplementary information
