# Supplementary material for: Coronary circulation enhances the aerobic performance of wild Pacific salmon
Source: J Exp Biol. 2024 Jul 12;227(20):jeb247422. doi: 10.1242/jeb.247422 (PMC11418299; doi:10.1242/jeb.247422)
Supplement: Supplementary information [file jexbio-227-247422-s1.pdf]

Table S1. Statistical results for metabolic rates measured in the coho salmon (*Oncorhynchus kisutch)* from the two-way ANOVA with treatment (sham-operated and coronary-ligated), sex (male or female), and its interaction as fixed effects. Due to a lack of interaction, the interaction was dropped from the final analysis and the statistics are reported for Treatment and Sex without the interaction included in each model.

Metric	Treatment			Sex			Treatment*Sex		
	df	F	P	df	F	P	df	F	P
Resting metabolic rate	1	0.032	0.860	1	4.661	**0.040**	1	0.218	0.644
Maximum metabolic rate	1	5.631	**0.025**	1	5.718	**0.025**	1	0.796	0.380
Absolute aerobic scope	1	5.151	**0.034**	1	2.410	0.136	1	0.129	0.723
Factorial aerobic scope	1	5.331	**0.032**	1	0.214	0.649	1	0.054	0.819

Two-way ANOVA results with df = degrees of freedom, F = F-value, P = P-value. Significant P-values are bolded.

Table S2. Linear models with treatment (sham-operated and coronary-ligated), sex (male or female) and timepoint (0, 15, and 60 min post-exercise and at rest [and after CT_{max} for blood metrics]) as fixed effects and fish ID as a random effect and associated results of model selection (BIC).

Metric	Equation	df	BIC	ΔBIC
	timepoint + treatment + sex + (1|id)	9	439.69	0.00
	timepoint + treatment * sex + (1|id)	10	444.02	4.33
MO_2 1 h recovery	timepoint * treatment + sex + (1|id)	13	452.95	13.26
	timepoint * sex + treatment + (1|id)	13	453.09	13.40
	timepoint * treatment * sex + (1|id)	22	484.98	45.29
	timepoint + treatment + sex + (1|id)	8	687.43	0.00
	timepoint + treatment * sex + (1|id)	9	691.42	3.99
PvO_2 1 h recovery	timepoint * treatment + sex + (1|id)	11	694.50	7.06
	timepoint * sex + treatment + (1|id)	11	700.38	12.95
	timepoint * treatment * sex + (1|id)	18	718.10	30.67
	timepoint + treatment + sex + (1|id)	9	717.14	0.00
	timepoint + treatment * sex + (1|id)	10	721.47	4.33
Plasma lactate	timepoint * sex + treatment + (1 |id)	13	729.03	11.88
	timepoint * treatment + sex + (1|id)	13	729.94	12.80
	timepoint * treatment * sex + (1|id)	22	760.25	43.11
	timepoint * sex + treatment + (1 |id)	13	76.77	0.00
	timepoint + treatment + sex + (1|id)	9	92.10	15.33
Plasma glucose[#]	timepoint + treatment * sex + (1|id)	10	96.85	20.07
	timepoint * treatment + sex + (1|id)	13	102.83	26.06
	timepoint * treatment * sex + (1|id)	22	106.24	29.46
	timepoint + treatment + sex + (1|id)	9	478.32	0.00
	timepoint + treatment * sex + (1|id)	10	481.94	3.62
Plasma potassium	timepoint * treatment + sex + (1|id)	13	491.70	13.39
	timepoint * sex + treatment + (1 |id)	13	495.62	17.30
	timepoint * treatment * sex + (1|id)	22	522.96	44.65
	timepoint + treatment + sex + (1|id)	9	927.04	0.00
	timepoint + treatment * sex + (1|id)	10	930.66	3.63
Plasma sodium	timepoint * sex + treatment + (1 |id)	13	942.96	15.93
	timepoint * treatment + sex + (1|id)	13	945.63	18.60
	timepoint * treatment * sex + (1|id)	22	980.51	53.47
	timepoint + treatment + sex + (1|id)	9	242.50	0.00
	timepoint + treatment * sex + (1|id)	10	247.26	4.75
Plasma cortisol[#]	timepoint * sex + treatment + (1 |id)	13	256.81	14.31
	timepoint * treatment + sex + (1|id)	13	258.95	16.45
	timepoint * treatment * sex + (1|id)	22	289.21	46.70

Hematocrit	timepoint + treatment + sex + (1|id)	9	738.41	0.00
	timepoint + treatment * sex + (1|id)	10	742.15	3.74
	timepoint * sex + treatment + (1 |id)	13	750.51	12.10
	timepoint * treatment + sex + (1|id)	13	754.31	15.90
	timepoint * treatment * sex + (1|id)	22	780.02	41.61

Represented are model formulas with random effects of fish id (1|id) and the resulting BIC model selection. df = degrees of freedom, BIC = Bayesian Information Criterion, ΔBIC = BIC(model) - BIC(min BIC value). # indicates the parameter is log-corrected.

Journal of Experimental Biology • Supplementary information

Table S3. Statistical results for blood metrics measured in the coho salmon (*Oncorhynchus kisutch)* from the repeated measures ANOVA with treatment (sham-operated and coronary-ligated), sex (male or female) and timepoint (0, 15, and 60 min post-exercise, at rest, and after CT_{max}) as fixed effects and fish ID as a random effect. The selected model is based on BIC (Table S1).

Blood Metric	Treatment			Sex			Timepoint			Timepoint*Sex		
	df	χ^2	P	df	χ^2	P	df	χ^2	P	df	χ^2	P
Lactate	1	0.700	0.404	1	0.740	0.390	4	204.393	**< 0.001**	NA	NA	NA
Glucose	1	0.527	0.468	1	0.811	0.368	4	31.301	**< 0.001**	4	41.952	**< 0.001**
Potassium	1	0.045	0.833	1	1.267	0.260	4	42.001	**< 0.001**	NA	NA	NA
Sodium	1	1.466	0.226	1	3.875	**0.049**	4	25.377	**< 0.001**	NA	NA	NA
Cortisol	1	0.238	0.626	1	41.528	**< 0.001**	4	136.963	**< 0.001**	NA	NA	NA
Hematocrit	1	0.084	0.772	1	1.338	0.247	4	69.653	**< 0.001**	NA	NA	NA

Repeated measures ANOVA results with df = degrees of freedom, χ^2 = chi-squared-value, P = P-value. Significant P-values are bolded.

Journal of Experimental Biology • Supplementary information
